# Safety and efficacy of the choline analogue SAR97276 for malaria treatment: results of two phase 2, open-label, multicenter trials in African patients

**DOI:** 10.1186/s12936-017-1832-x

**Published:** 2017-05-04

**Authors:** Jana Held, Christian Supan, Carmen L. Ospina Salazar, Halidou Tinto, Léa Nadège Bonkian, Alain Nahum, Ali Sié, Salim Abdulla, Cathy Cantalloube, Elhadj Djeriou, Marielle Bouyou-Akotet, Bernhards Ogutu, Benjamin Mordmüller, Andrea Kreidenweiss, Mohamadou Siribie, Sodiomon B. Sirima, Peter G. Kremsner

**Affiliations:** 1 Institut für Tropenmedizin, Universitätsklinikum Tübingen, Wilhelmstraße 27, 72074 Tübingen, Germany; 2 Centre de Recherches Médicales de Lambaréné, Lambaréné, Gabon; 3 German Centre for Infection Research, Partner Site Tübingen, Tübingen, Germany; 4 Centre Muraz–IRSS, Bobo-Dioulasso, Burkina Faso; 5 Centre de Recherche Entomologique de Cotonou, Cotonou, Benin; 6 Centre de Recherche en Santé de Nouna, Nouna, Burkina Faso; 7 Ifakara Health Research Center, Bagamoyo, United Republic of Tanzania; 8 Sanofi Research and Development, Chilly-Mazarin, France; 9 Département de Parasitologie, Mycologie, Médecine Tropicale, Université des Sciences de la Santé, Libreville, Gabon; 10 KEMRI Walter Reed Project, Kisumu, Kenya; 11 Groupe de Recherche Action en Santé, Ouagadougou, Burkina Faso; 12 Centre National de Recherche et de Formation sur le Paludisme, Ouagadougou, Burkina Faso

**Keywords:** Malaria, *P. falciparum*, SAR97276A, Choline analogue, Phase 2, Africa

## Abstract

**Background:**

Malaria remains one of the most important infectious diseases. Treatment options for severe malaria are limited and the choline analogue SAR97276A is a novel chemical entity that was developed primarily as treatment for severe malaria. Before starting clinical investigations in severely ill malaria patients, safety and efficacy of SAR97276A was studied in patients with uncomplicated malaria. Here, we summarize two open-label, multi-center phase 2 trials assessing safety and efficacy of parenterally administered SAR97276A in African adults and children with falciparum malaria.

**Results:**

*Study 1* was conducted in Burkina Faso, Gabon, Benin and Tanzania between August 2008 and July 2009 in malaria patients in an age de-escalating design (adults, children). A total of 113 malaria patients received SAR97276A. Adults were randomized to receive a single dose SAR97296A given either intramuscularly (IM) (0.18 mg/kg) or intravenously (IV) (0.14 mg/kg). If a single dose was not efficacious a second adult group was planned to test a three dose regimen administered IM once daily for 3 days. Single dose SAR97276A showed insufficient efficacy in adults (IM: 20 of 34 cured, 59%; and IV: 23/30 cured, 77%). The 3-day IM regimen showed acceptable efficacy in adults (27/30, 90%) but not in children (13/19, 68%). SAR97276A was well tolerated but no further groups were recruited due lack of efficacy. *Study 2* was conducted between October 2011 and January 2012 in Burkina Faso, Gabon and Kenya. SAR97276A administered at a higher dose given IM was compared to artemether–lumefantrine. The study population was restricted to underage malaria patients to be subsequently enrolled in two age cohorts (teenagers, children). Rescue therapy was required in all teenaged malaria patients (8/8) receiving SAR97276A once daily (0.5 mg/kg) for 3 days and in 5 out of 8 teenaged patients treated twice daily (0.25 mg/kg) for 3 days. All patients (4/4) in the control group were cured. The study was stopped, before enrollment of children, due to lack of efficacy but the overall safety profile was good.

**Conclusions:**

Monotherapy with SAR97276A up to twice daily for 3 days is not an efficacious treatment for falciparum malaria. SAR97276A will not be further developed for the treatment of malaria.

*Trial registration* at clinicaltrials.gov: NCT00739206, retrospectively registered August 20, 2008 for Study 1 and NCT01445938 registered September 26, 2011 for Study 2.

**Electronic supplementary material:**

The online version of this article (doi:10.1186/s12936-017-1832-x) contains supplementary material, which is available to authorized users.

## Background

Malaria caused by the protozoan parasites of the genus *Plasmodium* remains one of the most important infectious diseases. *Plasmodium falciparum* is the species responsible for nearly all severe malaria cases and deaths [1]. Treatment of uncomplicated as well as severe malaria heavily relies on one class of compounds—artemisinin derivatives. Starting in 2010 WHO has recommended parenteral artemisinins (artesunate as first-line treatment) also as treatment for severe malaria. Besides quinine no other substances for treatment of severe malaria are available. Reduced sensitivities against artemisinins [2] and the adverse event of delayed hemolytic anemia after parenteral artesunate [3] call for the development of new compounds. This is especially important for the treatment of severe malaria, where the development pipeline is particularly scarce [4].

SAR97276A (CAS number 321915-72-4) is a bis-thiazolium-dibromide whose precursor was synthesized in a series of choline analogues which act via interference with the choline metabolism of *P. falciparum* parasites and lead to the inhibition of phospholipid biosynthesis [5] and also interferes with heme-detoxification by binding to ferriprotoporphyrin IX [6]. Chemical optimization of choline analogues led to SAR97276A [7] which showed powerful in vitro-activity against *P. falciparum* laboratory and clinical isolates [5, 7]. It was also highly efficacious in a malaria mouse and monkey model [5, 7, 8]. SAR97276A was less active when given orally (curative 4-day dose in the mouse model 20–45 mg/kg/day orally; and 1 mg/kg/day parenterally) and was therefore administered parenterally.

SAR97276A was well tolerated in three phase 1 clinical trials evaluating single doses of 0.2–12.8 mg given intravenously (IV) to 52 participants, and single doses of 3.2–36 mg given intramuscularly (IM) to 48 participants, compared to 32 placebo controls (unpublished data owned by Sanofi). Multiple doses (3 days) were given in two phase 1 trials to 27 participants IV at doses from 4.3 to 12.8 mg and to 36 participants IM at doses of 12.5–36 mg compared to 21 placebo controls. Terminal half-life after single IM administration of 36 mg was 5.5 h in plasma and 73 h in whole blood. Adverse events (AEs) at the highest IV-single and multiple dose were accommodation disorders of the eye, blurred vision and gastrointestinal disorders (esophageal spasms); two participants had AEs of severe intensity displayed by severe abdominal spasm after receiving multiple doses of 12.8 mg IV. The highest administered dose was 36 mg given IM for 3 days. The highest tolerated dose for twice daily IM administration for 3 days was 18 mg, although one participant had an AE with severe intensity complaining of ophthalmologic problems including a temporary decrease in visual acuity that were considered to be drug related after receiving multiple administrations of 18 mg twice daily.

To develop SAR97276A for parenteral treatment of severe malaria, safety and efficacy was first assessed in uncomplicated falciparum malaria patients. Here, we summarize two open-label, adaptive, multi-center phase 2 studies of SAR97276A conducted in patients with uncomplicated malaria. Study 1 followed an adaptive, age de-escalating trial design aiming to progressively assess SAR97276A first in adults and in children with uncomplicated malaria, and then in children with severe malaria—provided that threshold criteria of efficacy were met at each cohort level together with a satisfactory safety profile. Due to lack of efficacy in children with uncomplicated malaria in Study 1, a follow up study (Study 2) was designed to evaluate a higher dose of SAR97276A at two different regimens (once daily or twice daily, each for 3 days) for safety and efficacy in pediatric patients with uncomplicated malaria.

## Methods

### Trial design summary

Study 1 and Study 2 followed both an open-label, adaptive, multi-center phase 2 trial design to assess safety and efficacy of SAR97276A in malaria patients of different age groups including adults (only Study 1) and children (both studies). Both studies were hospital-based. Study 1 was performed in Burkina Faso (three sites: Nouna, Bobo-Dioulasso, Ouagadougou), Benin (Cotonou), Gabon (Lambaréné), and Tanzania (Bagamoyo) between August 2008 and July 2009. Study 2 was done in Burkina Faso (Nouna, Bobo-Dioulasso, Ouagadougou), Gabon (Libreville), and Kenya (Ahero) from October 2011 to January 2012.

#### Study 1

**Fig. 1:**
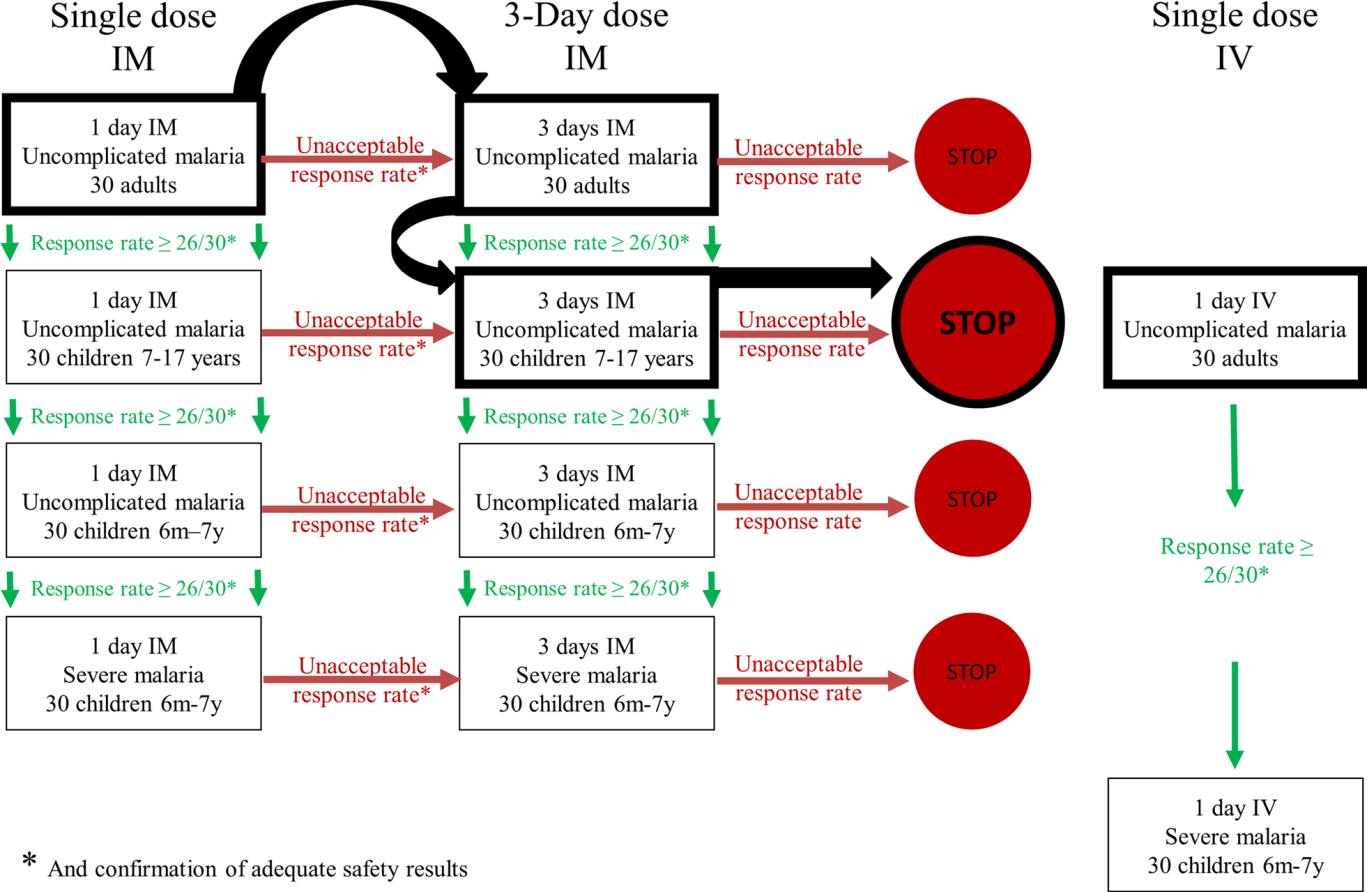
Study design and decision tree of Study 1. *Bold lines* and *black arrows* indicate the steps that have actually been followed during the trial

The study was designed to enroll up to 210 malaria patients in sequential cohorts. A staggered trial design was planned to assess a single, IM dose of SAR97276A (0.18 mg/kg, or 12.5 mg fixed dose) first in adults and then progressively in children (age 7–17 years) and young children (age 0.5–7 years). Age de-escalation of the study was only allowed if threshold response rate in the respective study cohort was met (26 patients out of 30 patients need to have met the 4 criteria of the primary endpoint at 72 h plus adequate safety results). If efficacy criteria were not met but safety was acceptable, SAR97276A would be tested as a 3-day IM regimen (3 injections, 24 h apart) in an additionally recruited 30 patients of the respective cohort. Subsequently, in case of acceptable safety and efficacy, all following cohorts were treated with the 3-day IM regimen. In parallel, SAR97276A was planned to be tested as a single, IV dose (0.14 mg/kg or 10 mg fixed dose) first in adults and then in young children with severe malaria, if threshold response rate was met. An overview of this adaptive study design can be seen in Fig. 1 and Table 1.

**Table 1:** Key characteristics of Study 1 and Study 2

	Study 1	Study 2
Countries	Benin, Burkina Faso, Gabon, Tanzania	Burkina Faso, Gabon, Kenya
N (planned)	210	180
N (pursued)	123	20
Age cohorts (years)	Adults (18–65)	Teenager (12–17)
	Children (7–17)	Children (2–11)^a^
	Young children (0.5–6)^a^	
Parasitemia at inclusion	Adults: >100 parasites/µL	>2000 parasites/µL
	Children: >1000 parasites/µL	
Severe malaria	Planned for young children cohort	No
SAR97276A treatment	1 day intramuscular 0.18 mg/kg (max. 12.5 mg)	3 days intramuscular once daily 0.5 mg/kg (max. 36 mg)
	3 days intramuscular 0.18 mg/kg (max. 12.5 mg)	
	1 day intravenous 0.14 mg/kg (max. 10 mg)	3 days intramuscular twice daily 0.25 mg/kg per dose (max. 18 mg per dose)
Control treatment	None	Artemether–lumefantrine

^a^These groups were not enrolled when study was conducted

#### Study 2

The study was designed to enroll up to 180 participants. A randomized, controlled, age de-escalating trial design was applied in Study 2 to assess SAR97276A as a 3-day regimen administered IM to underage malaria patients. Study participants were randomized to receive SAR97276A either once daily (0.5 mg/kg or 36 mg fixed dose) for 3 days or twice daily (0.25 mg/kg or 18 mg fixed dose per dose) for 3 days or artemether–lumefantrine (on admission, 8, 24, 36, 48 and 60 h later) in a 2:2:1 ratio. The study was planned to be conducted first in teenagers (age 12–17 years) and then in children (age 2–11 years). In case that exposure to SAR97276A (Cmax and AUC) measured in children was below the level of exposure of teenagers and the safety profile was satisfactory, an additional cohort of children (age 2–11 years) would have been enrolled to receive a higher dose to reach comparable exposure across age groups.

### Participants

#### Inclusion and exclusion criteria

General criteria for enrollment of study participants largely overlapped between Study 1 and Study 2. Detailed criteria are described per study in the supplement (see Additional file 1).* General inclusion criteria* *P. falciparum* infection diagnosed by Giemsa stained thick blood smear and fever (≥38 °C) or history of fever within the last 24 h, signed informed consent, in case of minors signed informed consent of parents/legal guardian, for Study 2 signed assent for children ≥12 years.* Exclusion criteria* Treatment with an antimalarial in the preceding 72 h of participant screening, severe concomitant disease, severe malnutrition, severe malaria, impossibility to be hospitalized and followed up, staff member or relative of staff member, parasitemia >100,000 parasites/µL blood, participation in another clinical trial within last 3 months, treatment (within 3 weeks) with cytochrome P450 inhibitor, pregnancy, breast-feeding, women not protected by birth control methods.

#### Age groups

##### Study 1

The following age cohorts were planned to be enrolled: (i) Adults aged 18–65 years, body weight ≥50 kg, and uncomplicated malaria with *P. falciparum* ≥100 parasites/µL; (ii) children aged 7–17 years, and uncomplicated malaria with *P. falciparum* ≥1000 parasites/µL, and (iii) young children aged of 0.5–7 years with uncomplicated malaria (*P. falciparum* count ≥1000 parasites/µL) and as an additional group with symptoms or signs of severe malaria according to WHO [9]. Adults received SAR97276A either by IM (study group 1A and 1B) or by IV route (study group 1C). Children received SAR97276A only by IM injection (study group 1D). Young children (0.5–7 years) both with uncomplicated and with severe malaria were not included as Study 1 was stopped before reaching these cohorts due to lack of efficacy.

##### Study 2

Study 2 only enrolled uncomplicated malaria patients with *P. falciparum* ≥2000 parasites/µL. Two age groups were planned: Teenager aged 12–17 years and children aged 2–11 years. SAR97276A was planned to be administered only by IM injection. The children cohort was not pursued as Study 2 was stopped before progressing to these groups due to insufficient efficacy.

### Interventions

The investigational product was SAR97276A provided by Sanofi. In Study 1, SAR97276A was injected either via the IM route at doses of 0.18 mg/kg (up to the highest fixed dose of 12.5 mg) or via the IV route at doses of 0.14 mg/kg (up to a highest fixed dose of 10 mg). For details of SAR97276A preparation for clinical administration, see supplement (Additional file 2). In Study 2, SAR97276A was provided in a ready to use solution for injection containing 9 mg/mL in a 7 mL vial. If volume of injection exceeded 2 mL (depending on weight of participant), it was split in two injections. Artemether–lumefantrine was administered to the control group at the standard regimen.

### Randomization

In Study 1, patients were not randomized but directly entered in the treatment groups when inclusion/exclusion criteria were met. Before proceeding from one cohort to the next, safety and efficacy data were reviewed by a Data Monitoring Committee (DMC) and the following group was started only when predefined limits were met. In Study 2, randomization to treatment allocations followed a 2:2:1 ratio: either once daily for 3 days, twice daily for 3 days or to the comparator drug. Randomization was done by an interactive voice/web response system on day 1. The system generated the randomization lists of the patients and assigned treatment number and the respective treatment for the patient accordingly. Randomization was not stratified by site and the study was not-blinded. To compensate for this a DMC assessed safety and activity independently from investigator and sponsor.

### Study procedures

*Study duration and follow-up* In both studies, study duration was 28 days for each patient including a screening period of up to 12 h before inclusion into the study. Participants were hospitalized for at least 3 days (72 h) after the start of treatment. Follow-up visits were done on day 6, 8, 14 ± 2, 21 ± 2, 28 ± 2 (Study 1); and day 7, 14 ± 2, 21 ± 2, 28 ± 2 (Study 2). *Patient examination* Fever measurements and thick blood smears were done every 6 h until 72 h and at each follow-up visit. Parasitemia was determined microscopically by Giemsa stained thick blood smear by two trained microscopists blinded to each other. *Safety assessment* Safety analysis was based on AEs including vision assessment recorded from baseline until day 28 (in binocular conditions, for near and distant visual acuity, using standard optotypes appropriate for patient’s age and reading ability. Tests with standard corrective glasses (+3 and −3 diopters) were performed in case of visual adverse events, to assess whether vision improved or worsened with corrective glasses). Laboratory tests including hematology, biochemistry and urine analysis were done at baseline, day 2, 4, 28 (Study 1), or baseline, day 2, 4, 14, 28 (Study 2); vital signs were recorded at baseline, every 6 h until 72 h and on each follow up visit. An electrocardiogram (ECG) was done at baseline, day 4, 28 (Study 1), or baseline, 48 h, 72 h, day 28 (Study 2). For Study 2 respiratory parameters (respiratory rate, peak flow, efficiency of cough) were also checked at baseline and 30 min after first dose on day 1, 2 and 3.

Rescue therapy was initiated in case of clinical aggravation of recrudescent parasites in both studies, the exact description for each study can be seen in the supplement (Additional file 3). Pharmacokinetic analysis: Plasma samples were assayed using a validated liquid chromatography-mass spectrometry method with a limit of quantification of 1 ng/ml for SAR97276. The pharmacokinetic analysis was performed using a population pharmacokinetic modeling approach.

### Outcomes

*Definition of fever clearance* Fever was defined as temperature ≥38 °C. Fever clearance was the time interval between the start of SAR97276A administration and the first time point at which the rectal or tympanic temperature fell below 38 °C and remained below 38 °C for at least 24 h.

*Definition of general condition improvement* The symptoms were scored at baseline and every 6 h up to day 4 (72 h), including headache, fatigue, nausea-vomiting and abdominal pain. The symptoms were scored according to the following scale: absent -0-, mild -1-, moderate -2- and severe -3-. The total clinical score ranged between 0 and 12. The total score was compared between baseline and 48 h post start of SAR97276A administration. A reduction of at least 70% of the total score or a total ≤1 was required, with no severe (score 3) malaria related symptom to define the general condition improvement.

#### Study1

*Primary endpoint* was response to treatment defined by 4 different criteria: fever clearance at 48 h, general conditions improvement at 48 h, at least 90% parasite reduction at 72 h, and no need for rescue therapy. To proceed to the following cohort at least 26/30 patients had to be classified as responders (positive response to all 4 criteria). *Secondary endpoints* included parasite reduction measurement, time to 50 and 90% parasite reduction (PC50 and PC90), day 28 polymerase chain reaction (PCR) corrected cure rate and pharmacokinetics. To distinguish between new infections and recurrent infections, 100 µL of blood was collected at baseline and at day of recurrence on filter paper and polymorphisms in merozoite surface protein (MSP)1, MSP2 and glutamate rich protein [10] were evaluated by PCR.

#### Study 2

*Primary endpoint* consisted of two criteria: (1) parasite clearance at 72 h analyzed by thick blood smear and (2) measurement of parasite reduction ratio (PRR: parasitemia at baseline/parasitemia at 72 h).

Treatment emergent adverse events (TEAEs) were defined as AEs that developed or worsened during the treatment period until day 6 following the end of study treatment.

Definition of early and late treatment failure can be found in the supplement (see Additional file 3).

### Statistical analysis

Both trials were overseen by a DMC. The intention to treat population (ITT) comprised all patients included in the study who received at least one dose of SAR97276A (both studies), analysis was performed on all patients.

#### Study 1

Sample size was chosen empirically, comprising 30 participants per cohort. The different cohorts were recruited sequentially and the maximum expected sample size would have reached 210 patients, if all cohorts were recruited.

The 72 h response rate (positive response to all 4 criteria) and the exact 95% and 90% 2–sided confidence limit was calculated for each cohort. To proceed to the following cohort the acceptable response rate had to meet the upper two sided 90% CL for a response rate ≥95%, reflected by at least 26/30 responders. PC50 and PC90 were calculated by Kaplan–Meier method.

#### Study 2

Sample size was calculated based on the parasite reduction ratio (PRR). Details are described in the supplementary information (see Additional file 4). PRR was calculated by: parasitemia at baseline/parasitemia at 72 h. For patients who prematurely discontinued study treatment the parasitemia at 72 h was replaced by last measurement of parasitemia or last measurement before rescue therapy for calculation of PRR at 72 h.

Parasite clearance time (PCT), PC50 and PC90 were calculated by Kaplan–Meier method. Interim analysis was to be performed following completion of each cohort.

## Results

A summary of the design of Study 1 and Study 2 is depicted in Table 1. Both studies were designed to enroll study participants in an age de-escalating design provided that efficacy and safety threshold criteria were met at each age level. In Study 1, SAR97276A was tested in 4 allocation groups as a single dose given IM to adults (Group 1A), as a 3-days regimen administered IM to adults (Group 1B), as a single dose injected IV to adults (Group 1C), and as a 3-day regimen administered IM to children (Group 1D). Study 2 enrolled only teenagers and SAR97276A was tested as a 3-days regimen administered IM either once daily (Group 2A) or twice daily (Group 2B). Control teenagers received artemether–lumefantrine (Group 2C).

The following detailed results were achieved in each study:

### Study 1

**Fig. 2:**
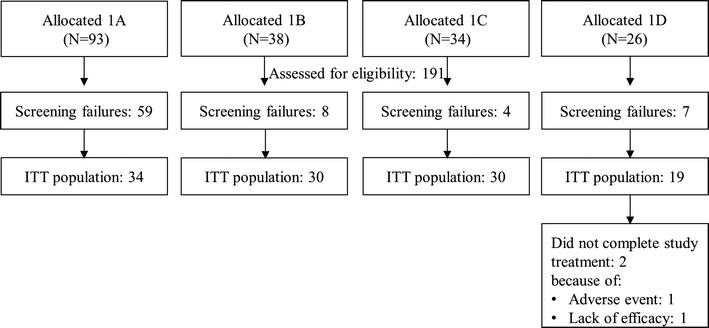
Study flow and inclusion of study participants (N) to SAR97276A allocation groups (Study 1). Group 1A: 1 day IM to adults; Group 1B: 3 days IM to adults; Group 1C: 1 day IV to adults; Group 1D: 3 days IM to children

In Study 1, a total of 113 malaria patients were treated with SAR97276A. All patients were available for efficacy and safety analysis. The study flow is shown in Fig. 2, baseline characteristics are presented in Table 2.

**Table 2:** Summary of key variables and baseline characteristics of Study 1 and Study 2

Group	Study 1				Study 2		
	1A	1B	1C	1D	2A	2B	2C
N	34	30	30	19	8	8	4
Benin (n)	0	0	0	2	0	0	0
Burkina Faso (n)	34	24	26	0	4	5	2
Gabon (n)	0	6	4	12	2	2	2
Tanzania (n)	0	0	0	5	0	0	0
Kenya (n)	0	0	0	0	0	1	0
Age (in years)^a^	39 (18–64)	35 (18–60)	37 (18–63)	9 (7–17)	14 (12–17)	14 (12–16)	13 (12–16)
Body temp. (°C)^a^	37.1 (35.9–39.4)	37.2 (36.5–38.9)	37.2 (36.3–38.3)	37.6 (36.8–40)	37.2 (36.9–39.9)	38.0 (36.2–40.3)	38.1 (37.0–38.8)
BMI in kg/m^2a^	21 (15–32)	23 (17–38)	21 (18–31)	16 (12–22)	17 (15–18)	17 (14–23)	18 (14–21)
Parasitemia (parasites/µL)^a^	688 (112–33,680)	646 (114–67,500)	518 (117–11,920)	44,000 (1115–99,200)	6318 (2105–69,155)	6080 (2016–31,400)	12,811 (2003–28,559)
Hemoglobin (g/L)^a^	129 (106–146)	117 (94–146)	132 (82–160)	102 (80–127)	112	117	126
Treatment response	20/34 (58.8%)	27/30 (90%)	23/30 (76.6%)	13/19 (68.4%)	0/8 (0%)	3/8 (37.5%)	4/4 (100%)
Patients with any TEAE	18 (52.9%)	18 (60%)	14 (46.7%)	11 (57.9%)	8 (100%)	5 (63%)	1 (25%)
Patients with any serious AE	3 (8.8%)	0	0	1 (5.3%)	4 (50%)	2 (25%)	0

^a^Median (Min–Max)

Study 1: Allocation of SAR97276A to the following groups: Group 1A: 1 day IM administration to adults; Group 1B: 3 days IM administration to adults; Group 1C: 1 day IV route to adults, Group 1D: 3 days IM administration to children. Study 2: SAR97276A allocation to the following groups: Group 2A: once daily for 3 days IM administration to teenager; Group 2B: twice daily for 3 days IM administration to teenager. Group 2C: artemether–lumefantrine control treatment to teenager

#### Efficacy assessment

**Fig. 3:**
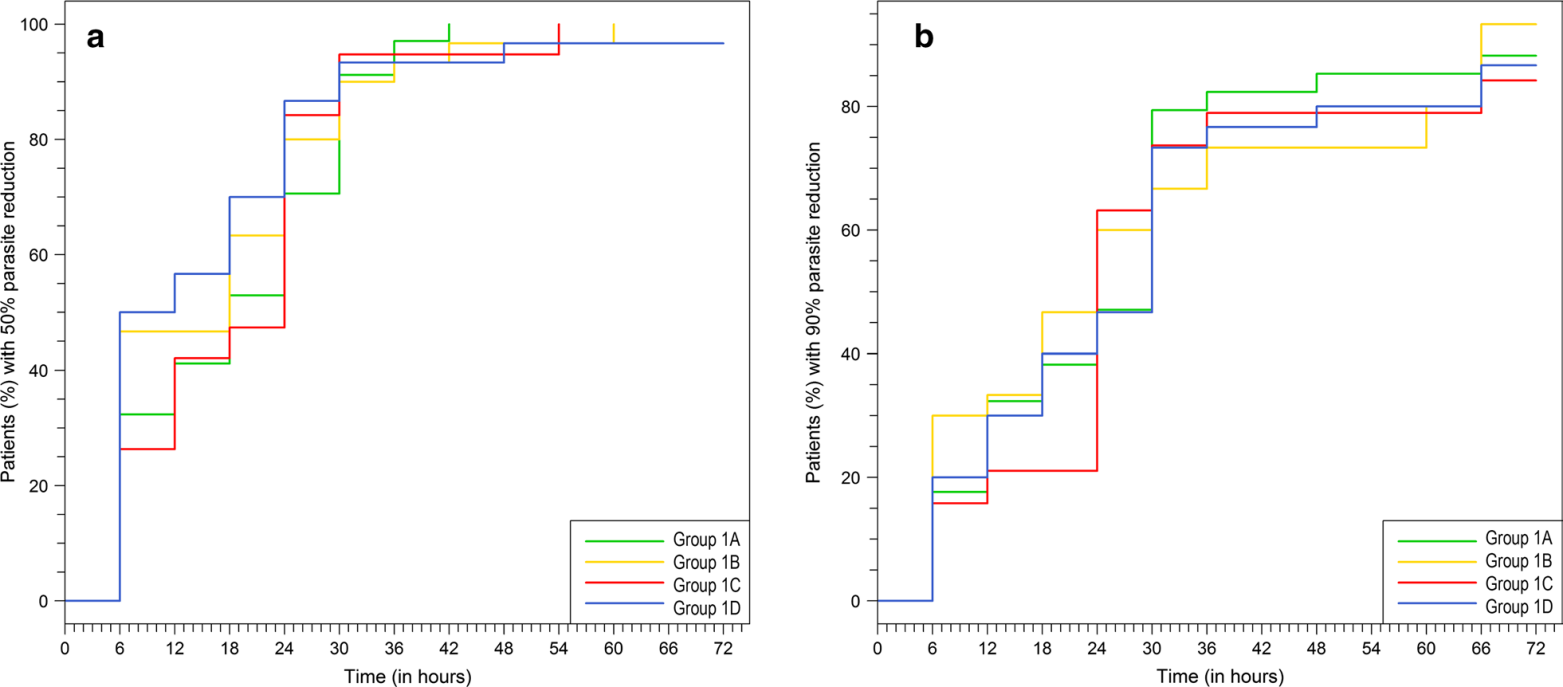
Kaplan Meier estimates of **a** the 50% parasite reduction and **b** the 90% parasite reduction (Study 1). Group 1A: 1 day IM to adults; Group 1B: 3 days IM to adults; Group 1C: 1 day IV to adults, Group 1D: 3 days IM to children

When adults were treated with a single dose of SAR97276A either IM in Group 1A or IV in Group 1C, 20 out of 34 (20/34, 59%, accidental inclusion of 34 participants instead of 30) and 23/30 (77%) were cured at 72 h, respectively (Table 3). This was below the pre-specified efficacy threshold (26 patients cured out of 30 at 72 h) and the regimen was changed to once daily for three days in the subsequent cohorts. When given IM for 3 days the response rate at 72 h for adults was 27/30 (90%) and 13/19 (69%) in children aged 7–17 years. The results for the single response criteria (fever clearance, general condition improvement, parasite reduction, and rescue therapy) are depicted in Table 3. Due to the low response rate in the children’s cohort, the DMC recommended not to progress with the trial. Median time to 50 and 90% parasite reduction is outlined in Table 4. Kaplan–Meier estimates for 50 and 90% reduction rates were similar between groups and are given in Fig. 3a, b.

**Table 3:** Outcomes of criteria determining treatment success

Group	1A	N = 34	1B	N = 30	1C	N = 30	1D	N = 19	
Criteria	Success	Failure	Success	Failure	Success	Failure	Success	Failure	Not evaluable
Fever clearance	30 (88%)	4 (12%)	29 (97%)	1 (3%)	29 (97%)	1 (3%)	16 (84%)	1 (5%)	2 (11%)
General condition improvement	32 (94%)	2 (6%)	28 (93%)	2 (7%)	29 (97%)	1 (3%)	17 (90%)	1 (5%)	1 (5%)
Parasite reduction	23 (68%)	11 (32%)	28 (93%)	2 (7%)	23 (77%)	7 (23%)	14 (74%)	5 (26%)	0
Rescue therapy	29 (85%)	5 (15%)	29 (97%)	1 (3%)	29 (97%)	1 (3%)	15 (79%)	4 (21%)	0
Response rate for all 4 criteria	20/34 (59%)		27/30 (90%)		23/30 (77%)		13/19 (69%)		
90% CI^a^	(0.43–0.73)		(0.76–0.97)		(0.61–0.89)		(0.47–0.85)		
95% CI^a^	(0.41–0.75)		(0.73–0.98)		(0.58–0.90)		(0.43–0.87)		

In the upper part of the table the four different criteria determining treatment success at 72 h are shown for the different groups. In the lower part the overall response rate together with the 90 and 95% confidence interval is shown. A positive response can only be achieved if all 4 criteria had a positive response at 72 h. (Study 1)

Group 1A: 1 day IM to adults; Group 1B: 3 days IM to adults; Group 1C: 1 day IV to adults; Group 1D: 3 days IM to children

^a^Confidence interval for the response rate for all 4 criteria

**Table 4:** Median time to 50 and 90% parasite reduction and recurrent infections up to day 28 (Study 1)

Group	1A	1B	1C	1D
N	34	30	30	19
Median time (h) to 50% parasite reduction (95% CI)	18.0 (12.0–24.0)	18.0 (6.0–24.0)	9.0 (6.0–18.0)	24.0 (12.0–24.0)
Median time (h) to 90% parasite reduction (95% CI)	30.0 (18.0–30.0)	24.0 (12.0–36.0)	30.0 (18.0–30.0)	24.0 (24.0–30.0)
Recurrences	14	13	15	8
PCR data available	12/14	11/13	10/15	8/8
New infection	9 (75%)	6 (54.5%)	6 (60%)	0
Recrudescences	3 (25%)	5 (45.5%)	4 (40%)	8 (100%)

Recurrent infections were only analyzed in patients who were judged as successfully treated after 72 h

Group 1A: 1 day IM to adults; Group 1B: 3 days IM to adults; Group 1C: 1 day IV to adults; Group 1D: 3 days IM to children

To evaluate the long-term antiplasmodial effect of SAR97276A recurrent infections until day 28 were assessed and genotyped to distinguish between new and recurrent infections. In total 50 recurrences occurred in the successfully treated patients until day 28. Of these recurrences 41 could be genotyped by PCR, revealing that about half of them were new infections (n = 21) (Table 4).

#### Safety assessment

Out of 113 patients 61 (54%) experienced a TEAE, the incidence was comparable between the cohorts. Most frequent TEAEs were nervous system disorders including headache and dizziness, followed by gastrointestinal disorders and vascular disorders, no administration site disorders were recorded. Hypotension was more frequent in the IV cohort: 7/30 patients in the Group 1C had hypotension (systolic and diastolic) that was judged as related to the study treatment whereas only 1/34 patients in Group 1A, 1/30 in Group 1B and none in the children’s cohort (Group 1D) experienced hypotension. All patients recovered without sequelae. Frequency and distribution of most frequent TEAEs are displayed in Table 5. No ophthalmologic disorders possibly related to SAR97276A were recorded.

**Table 5:** Treatment emergent adverse events, TEAEs (Study 1)

Group	1A	1B	1C	1D
N	34	30	30	19
Patients with any TEAE	18 (52.9%)	18 (60%)	14 (46.7%)	11 (57.9%)
Patients with any serious AE	3 (8.8%)	0	0	1 (5.3%)
Patients who did not complete study treatment	0	0	0	2
Headache	4 (11.8%)	4 (13.3%)	3 (10%)	3 (15.8%)
Dizziness	1 (2.9%)	1 (3.3%)	2 (6.7%)	0
Convulsion	0	0	0	1 (5.3%)
Paraesthesia	0	1 (3.3%)	0	0
Vertigo	2 (5.9%)	2 (6.7%)	0	0
Gastrointestinal disorders	3 (8.8%)	7 (23.3%)	1 (3.3%)	5 (26.3%)
General Disorders/Administration site disorders	3 (8.8%)	1 (3.3%)	0	1 (5.3%)
Hypertension	0	1 (3.3%)	0	0
Hypotension	1 (2.9%)	1 (3.3%)	7 (23.3%)	0
Thrombocytopenia	0	0	0	2 (10.5%)
Bradycardia	1 (2.9%)	0	0	0
Tachycardia	0	1 (3.3%)	0	0
Cough	2 (5.9%)	2 (6.7%)	1 (3.3%)	1 (5.3%)
Eyelid pruritus	0	0	0	1 (5.3%)
Pruritus	0	1 (3.3%)	0	0
Urticaria	1 (2.9%)	0	0	0
Arthralgia	1 (2.9%)	3 (19%)	1 (3.3%)	0
Neck pain	2 (5.9%)	1 (3.3%)	0	0
Cytolytic hepatitis	1 (2.9%)	0	0	0
Malaria	2 (5.9%)	0	0	0
Pneumonia	1 (2.9%)	0	0	0
Rhinitis	2 (5.9%)	0	0	0

Group 1A: 1 day IM to adults; Group 1B: 3 days IM to adults; Group 1C: 1 day IV to adults; Group 1D: 3 days IM to children

In total, four patients had a serious adverse event (SAE,) three patients in Group 1A (2 hospitalizations due to severe malaria and 1 cytolytic hepatitis of mild intensity) and one patient in Group 1D, the children group. The patient in Group 1A diagnosed with cytolytic hepatitis three days after having received the study medication experienced ALT increase of 4.5 × upper limit than normal (ULN), AST increase of 11 × ULN and total bilirubin of ≥1.5 × ULN. No malaria parasites were found at this time point. The one patient with severe malaria of Group 1A was admitted to the hospital 3 days after administration of single dose of SAR97276A, with fever (40.2 °C), shivering and vomiting and a parasitemia of 24,413 parasites/μL. After quinine treatment IV the patient felt better and was discharged the next day with no detectable parasites on the smear and with oral quinine treatment. The other patient with severe malaria was admitted to hospital 48 h after single injection with study medication with fever (39.8 °C), chills and convulsion and a parasitemia of 10,640 parasites/µL. The patient received quinine IV and all symptoms were resolved on the same day, parasites were completely cleared 3 days later. The patient in the children Group 1D experienced repeated convulsions of moderate intensity that led to discontinuation of study medication. All SAE-patients recovered without sequelae. None of the SAEs were regarded as related to SAR97276A by the investigators.

Two children (Group 1D) receiving the 3-day IM regimen did not complete study treatment. One patient experienced repeated convulsions (see above) and one showed early clinical and parasitological failure. No deaths occurred in the study. Hematology, electrocardiography and vital signs can be found in the supplement (see Additional file 5).

### Study 2

**Fig. 4:**
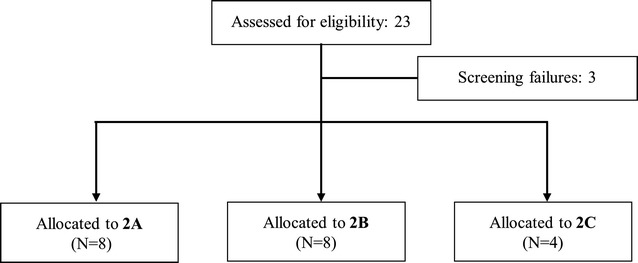
Study flow (Study 2). Group 2A: 3 days of once daily IM injection SAR97276A (0.5 mg/kg or 36 mg). Group 2B: 3 days of 2 daily IM injections SAR97276A (every 12 h 0.25 mg/kg or 18 mg). Group 2C: 3 days oral ACT according to the countries recommendations, i.e. artemether–lumefantrine

In **Study 2,** a total of 16 teenagers received SAR97276A. Based on low response rates to SAR97276A, phase 2 study was limited to teenagers (aged 12–17 years) randomized to the following allocation groups: Group 2A (8 teenagers received SAR97276A once daily IM for 3 days), Group 2B [8 teenagers received SAR97276A twice daily IM for 3 days (same cumulative dose as the once daily dose)] or Group 2C (4 teenagers received artemether–lumefantrine treatment). See Fig. 4 for study flow. Baseline characteristics were similar between the groups (Table 2).

#### Efficacy assessment

All (8/8) patients in Group 2A and 5/8 in Group 2B received rescue treatment during the trial, whereas all patients (4/4) of the control Group 2C were cured. 7/8 patients in Group 2A were classified as treatment failures (4 early and 3 late failures) and one patient having hepatitis B could not be evaluated according to the predefined definitions. 5/8 participants were treatment failures in Group 2B group (4 early and 1 late failure), none in the control Group 2C group was classified as treatment failure. Median PRR at 72 h was 22.5 (0.4–6280.0) and 12.0 (0.3–83.1) in Group 2A and Group 2B, respectively. Group 2C patients had a PRR of 2562.2 (400.6–5711.8). Increase of parasitemia between 6 and 12 h after the start of treatment was observed in Group 2A, which was not seen in the other groups. Median parasite clearance time was 144 h (CI 30–144 h) for Group 2A, 144 h (CI 30–144 h) for 2B, and 27 h (CI 18–66 h) for the 2C group. The trial was stopped after recruitment of the first 16 participants due to lack of efficacy.

#### Safety assessment

The most frequent TEAE was malaria in both SAR97276A treatment groups and neutropenia in Group 2B followed by gastrointestinal disorders, especially diarrhea, in Group 2A (Table 6). No administration site disorders were recorded. Other than one case of severe hepatitis B in the Group 2A, all other TEAEs were of mild or moderate intensity. Neutropenia, heartburn, abdominal pain and blurred vision were assessed as possibly related to SAR97276A. Four patients with a TEAE discontinued study treatment and received rescue treatment (Table 6). Of those, two had neutropenia on day 2 (0.99 and 0.91 giga/L) that returned to normal values at 72 h after treatment start. The two other patients discontinued treatment because of mild persistent malaria and hepatitis B. There were 6 patients who had a SAE, all were in the SAR97276A treatment groups (Table 6) but judged as not related to study treatment. Of those six patients, five had a SAE with persistence of uncomplicated malaria. The sixth patient who had hepatitis B and jaundice had abnormal liver function test values already at inclusion (ALT 33 × ULN, AST 21 × ULN and total bilirubin 3.94 × ULN); the patient was positive for HBs antigen on day 4 and for anti-HBc IgG and IgM on day 21, he recovered from clinical symptoms of hepatitis by day 28.

**Table 6:** Treatment emergent adverse events, TEAEs (Study 2)

Group	2A	2B	2C
N	8	8	4
Patients with any TEAE	8 (100%)	5 (63%)	1 (25%)
Patients with any serious AE	4 (50%)	2 (25%)	0
Patients with TEAE leading to treatment discontinuation	2 (25%)	2 (25%)	0
Malaria	7 (88%)	4 (50%)	0
Hepatitis B	1 (13%)		
Neutropenia	0	2 (25%)	0
Decreased appetite	0	1 (13%)	0
Headache	0	0	1 (25%)
Lacrimation increased	1 (13%)	0	0
Vision blurred	1 (13%)	0	0
Vertigo	1 (12.5%)	0	o
Gastrointestinal disorders	4 (50%)	1 (13%)	0
Jaundice	1 (13%)	0	0
Pyrexia	0	0	1 (25%)

Electrocardiogram parameters can be found in the supplement (see Additional file 6).

### Pharmacokinetics of Study 1 and Study 2

The pharmacokinetics of SAR97276 were best described by a three-compartment model, with a first order absorption (ka), with interindividual variability on clearances and central volume of distribution.

Descriptive statistics of the individual pharmacokinetic parameters for the different groups are presented in Table 7. Children presented a higher median clearance (0.393 vs. 0.275 L/h/kg) and a smaller median central volume of distribution V2 (0.217 vs. 0.270 L/kg) compared to adults. Even though dosing was weight adjusted, after IM dosing children showed a lower AUC but similar Cmax when compared to adults. Inter-individual variability was low (<25%) based on Cmax and AUC whatever the populations or the route of administration.

**Table 7:** Pharmacokinetics (Study 1 + Study 2)

	Study 1			Study 2	
	Adults IM (1A + 1B)	1C	1D	2A	2B^b^
n	64^c^	30	14	8	8
Cmax or Cmax,ss (ng/mL)^a^	355 ± 51	390 ± 55	332 ± 91	862 ± 45	477 ± 91
AUC or AUC0-24,ss (ng.h/mL)^a^	674 ± 109	480 ± 92	479 ± 111	1470 ± 375	1400 ± 270

Pharmacokinetic parameters: AUC 0-24ss: AUC24 at steady state; Cmaxss: Cmax at steady state

^a^Mean and SD

^b^For the twice daily treatment group, the AUC0-24 at steady state was the AUC0-12 at steady state multiplied by 2

^c^Pooling patient data after 1 day and 3 day treatment since no accumulation of exposure is observed after repeated dosing

In Study 2 AUC values were similar between the two different regimens of SAR97276A and Cmax was approximately 1.8-fold lower in Group 2A. Interindividual variability of pharmacokinetic parameters was low.

## Discussion

Results of these two consecutive phase 2 studies showed that SAR97276A monotherapy is not efficacious to cure uncomplicated falciparum malaria in children. WHO recommends a cure rate of 95% assessed in clinical trials to advance clinical development of a new antimalarial towards licensure [11]. The primary endpoint following IM administration of SAR97276A for 3 days treatment met the criteria of efficacy in the adult population in Study 1 with 27/30 cured patients. However, this result could not be reproduced in children when the same dose as in the adults was given nor at the higher dose of SAR97276A in the following study (Study 2). Study 1 was terminated when in the children’s cohort 5/19 enrolled patients failed to meet the predetermined efficacy response criteria. Due to the acceptable safety profile and the promising efficacy in the adult cohort receiving the 3-day treatment a second study (Study 2) was conducted. Study 2 was designed to evaluate the activity of a higher dose of SAR97276A in children after confirming safety of this dose in phase 1 studies. Study 2 was also terminated prematurely as efficacy was not acceptable, reflected by the fact that most teenaged patients receiving SAR97276A required rescue therapy. The observed difference in efficacy between adults and children could be explained by the acquired level of immunity in adults, a feature that is well described from endemic areas [12]. Young children are not semi-immune and therefore cannot clear parasites. In addition, parasitemia as inclusion criteria was set on a lower threshold for adults (>100 parasites/µL) than for children. Indeed, in 19 out of 30 adults of Group 1B of Study 1 parasitemia was below 1000 parasites/µL at inclusion, a fact that might also influence the efficacy outcome. Another reason for the lower efficacy might be the different pharmacokinetic profile as children had a lower AUC when compared to adults. Differences in efficacies of the drug between children from Study 1 and 2 are more difficult to explain, as children in Study 2 were older (median 14 years) than in Study 1 (9 years). Children were recruited partly from different study sites and differences in exposure to malaria might explain the result as level of immunity can differ substantially between different age groups and geographic settings [13, 14]. In addition, in Study 2 occurrence of parasites until day 14 were judged as late treatment failure. In Study 1 efficacy was only assessed until 72 h as a primary endpoint and 3/13 successfully cured children (assessed after 72 h) had a recrudescence of the same parasite until day 14, explaining partly the difference in efficacy in children of the two studies.

SAR97276A showed a reasonable safety profile in both studies. Deterioration of malaria was indirectly due to SAR97276A as it was not efficacious enough to control parasitemia and malaria symptoms.

The new mechanism of action of SAR97276A could make it an interesting partner for combination therapy with another strong malaria medication. However, as oral availability is low it has to be given parenterally and is therefore not a good candidate compound for treatment of uncomplicated malaria as this should be treated orally. Compounds against severe malaria should have a rapid onset of action, which cannot be seen by SAR97276A in the current studies. SAR97276A does not show a post-treatment prophylactic effect reflected by recurrent as well as new infections until day 28. As a secondary endpoint the parasite clearance time of SAR97276A was evaluated. When compared to artemisinins, the licensed drug class with the fastest clearance of parasites [15, 16], SAR97276A showed a much slower parasite clearance, underlining that it is not at all a suitable compound for the treatment of severe malaria.

## Conclusion

SAR97276A will not be further developed. However, compounds acting against phospholipid biosynthesis remain potentially interesting with a mode of action different to other antimalarials currently in use.

## Additional files

**Additional file 1.** Detailed inclusion and exclusion criteria of Study 1 and Study 2.

**Additional file 2.** Preparation of the investigational product SAR9727A (Study 1).

**Additional file 3.** Definition of early and late treatment failure (Study 2).

**Additional file 4.** Details on sample size calculation (Study 2).

**Additional file 5.** Results of hematology, electrocardiography and vital signs (Study 1). **Table S1**. Electrocardiogram abnormalities during TEAE period (Study1).

**Additional file 6.** ECG parameters (Study 2). **Table S2**. Electrocardiogram abnormalities during TEAE period (Study2). **Table S3**. Respiratory parameters (Study 2).

## Abbreviations

ACT: artemisinin combination therapy
DMC: Data Monitoring Committee
ECG: electrocardiogram
*P. falciparum*: *Plasmodium falciparum*
PRR: parasite reduction ratio
PCT: parasite clearance time
SAE: serious adverse event
TEAE: treatment emergent adverse event
ULN: upper limit of normal
