# Supplementary material for: Safety and efficacy of the choline analogue SAR97276 for malaria treatment: results of two phase 2, open-label, multicenter trials in African patients
Source: Malar J. 2017 May 4;16:188. doi: 10.1186/s12936-017-1832-x (PMC5418711; doi:10.1186/s12936-017-1832-x)
Supplement: Supplementary file 1 — Additional file 1. Detailed inclusion and exclusion criteria of Study 1 and Study 2. [file 12936_2017_1832_MOESM1_ESM.docx]

**Additional file 1: Detailed inclusion and exclusion criteria of Study 1 and Study 2**

**Study 1:**

**Inclusion criteria of Study 1:**

| **General criteria:** | - Patients diagnosed with *P. falciparum* malaria microscopically confirmed in blood smear | | | | |
| --- | --- | --- | --- | --- | --- |
|  | - Fever or history of fever within the last 24 hours before administration: auricular or rectal temperature ≥38°C | | | | |
|  | - Signed informed consent by patient with legal majority | | | | |
|  | - Signed informed consent by parent/legal guardian of pediatric patient or patient with no legal majority | | | | |
|  | |  |  | | |
| **Specific criteria:** | | **Cohort 1:** | - Age: ≥18 years to ≤65 years | | |
|  | |  | - Body weight: ≥50 kg | | |
|  | |  | - Symptomatic uncomplicated malaria defined as asexual parasitemia: *P. falciparum* ≥100 parasites/μL | | |
|  | | **Cohort 2:** | **Group 1** | - Age: ≥ 7 years to ≤ 17 years |  |
|  | |  |  | - Symptomatic uncomplicated malaria defined as asexual parasitemia: *P. falciparum* ≥1,000 parasites/μL |  |
|  | |  | **Group 2** | **(not recruited)** |  |
|  | |  |  | - Age: ≥ 6 months to < 7 years |  |
|  | |  |  | - Symptomatic uncomplicated malaria defined as asexual parasitemia: *P. falciparum* ≥1,000 parasites/μL |  |
|  | | **Cohort 3:** | **(not recruited)** | | |
|  | |  | - ≥ 6 months to < 7 years | | |
|  | |  | - Symptoms or signs of severe malaria, defined according to WHO by the presence of one or more of the following criteria: | | |
|  | |  | Clinical manifestations: persistent vomiting, prostration, impaired consciousness, multiple convulsions, jaundice | | |
|  | |  | Laboratory abnormalities: severe anemia: hemoglobin <50 g/L, acidosis, renal impairment: serum creatinine level >264 μmol/L with urine output <400 mL/24 hours | | |
|  | |  | Hyperparasitemia: asexual parasitemia ≥250,000 parasites/μL in a high transmission setting | | |

**Exclusion criteria of Study 1:**

| - Treatment with an antimalarial agent within 72 hours of screening expected to interfere with the assessment of the safety and efficacy of SAR97276A (treatment with 1 single tablet of quinine or chloroquine administered within the previous 72 hours is not exclusionary, treatment with any other antimalarial agent is) | |
| --- | --- |
| - Severe concomitant disease (including concomitant infection) | |
| - Severe malnutrition | |
| - High grade severe malaria as defined by the presence of 1 of the following syndromes or clinical symptoms: | |
|  | - coma with Blantyre coma scale (BCS) <3 - circulatory collapse with systolic blood pressure < 50 mmHg - pulmonary edema (radiological) - abnormal bleeding, - haemoglobinuria - any sign of respiratory distress - hypoglycemia <2.2 mmol/L - severe metabolic acidosis (bicarbonates <15 mmol/L) or hyperlactaemia (lactate >5mmol/L). |
| - Impossibility to be hospitalized and followed up | |
| - Patient is the Investigator or any Sub-Investigator, research assistant, pharmacist, study coordinator, other staff or relative thereof directly involved in the conduct of the protocol | |
| - Specific to Cohort 1 and Cohort 2: asexual parasitemia: *P. falciparum* >100,000 parasites/μL | |
| - Exclusion criteria related to SAR97276A treatment | |
| - Participation in another clinical trial within the last 3 months or participation within a different cohort in this ACT10004 clinical trial | |
| - Previous treatment within 3 weeks prior to inclusion, and concomitant treatment with potent cytochrome P450 (CYP) 3A4 inhibitors or CYP3A4 inducers or CYP2D6 substrates | |
| - Pregnant or breast-feeding women | |
| - Women of childbearing potential not protected by an effective method of birth control for 2 weeks before the start of study drug treatment and 1 week after the end of study drug treatment, and/or who are unwilling or unable to be tested for pregnancy (pregnancy status should be checked by urine pregnancy testing prior to exposure to the investigational product) | |
| - Pregnant or breast-feeding women | |

**Study 2:**

**Inclusion criteria of Study 2:**

| **General criteria:** | - Patients diagnosed with *P. falciparum* malaria microscopically confirmed in blood smear at Day-1 visit | | |
| --- | --- | --- | --- |
|  | - Fever or history of fever within the last 24 hours before administration: auricular or rectal temperature ≥38,5°C | | |
|  | - Asexual parasitemia of ≥2 000 parasites/μL in blood smear at D-1 visit | | |
|  | - Signed informed consent by parent/legal guardian of pediatric patient. In addition, participants with capacity for writing will sign off an Assent Form. Patients with no capacity for writing will have the Assent Form read. In that case, an impartial witness will certify the document was read to the child | | |
|  | |  |  |
| **Specific criteria:** | | **Cohort 1:** | - Age: ≥12 years to ≤17 years |
|  | | **Cohort 2:** | **(not recruited)** |
|  | |  | - Age: ≥2 years to ≤11 years |

**Exclusion criteria of Study 2:**

| **Related to study methodology:** | - Participation in another clinical trial within the last 3 months or participation within a different cohort in this PDY11737 clinical trial |
| --- | --- |
|  | - Participation to previous trial with SAR97276 |
|  | - Documented history of adequate treatment with antimalarials expected to be effective within the preceding 72 hours (treatment with a single tablet of quinine or chloroquine within the previous 72 hours is acceptable) |
|  | - Severe concomitant disease (including concomitant febrile illnesses or infection) |
|  | - Any sign suggestive of severe malaria |
|  | - Requirement for concomitant treatment that could bias the primary evaluation |
|  | - Any condition that could interfere with the study assessments (including efficacy) especially for safety (e.g. blindness) |
|  | - Severe malnutrition |
|  | - Asexual parasitemia: *P. falciparum* > 100,000 parasites/μL in blood smear at D-1 visit - Uncooperative or any condition that could make the patient potentially non-compliant to the study procedures |
|  | - Impossibility to meet specific protocol requirements such as the impossibility to stay at hospital during the first 4 days of study participation or be followed up in ambulatory manner up to at least the 28th day inclusive |
|  | - Previous treatment within 3 weeks prior to inclusion, and concomitant treatment with potent CYP3A4 inhibitors or CYP3A4 inducers or CYP2D6 substrates or potent CYP2D6 inhibitors |
|  | - Patient is relative of the Investigator or of the Sub-Investigator, or of a study nurse directly involved in the conduct of the protocol |
|  | - Any technical/administrative reason that makes it impossible to randomize the patient in the study |
|  |  |
| **Related to the active comparator and/or mandatory background**  **therapies** | - Known serious adverse event reaction or hypersensitivity to ACTs or any contraindications from the positive control therapy or warning/precaution of use as defined in the respective National Product Labeling |
|  |  |
| **Related to the current knowledge of Sanofi compound** | - Pregnant or breast-feeding women (Cohort 1) - Women of childbearing potential (Cohort 1) not protected by effective contraceptive method of birth control, or not willing to use an effective contraceptive(s) method(s) for the duration of the study (e.g.: double barrier method), and/or who are unwilling or unable to be tested for pregnancy. Pregnancy status should be checked by serum or urine pregnancy testing prior to exposure to the investigational product. The need for an efficient birth control method will be reminded to the patient and the patient’s legal guardian(s) by the investigator |
|  | - CPK above 3 ULN |
|  | - Underlying hepatobiliary disease or ALT>3 ULN |
