# Supplementary material for: Safety and efficacy of the choline analogue SAR97276 for malaria treatment: results of two phase 2, open-label, multicenter trials in African patients
Source: Malar J. 2017 May 4;16:188. doi: 10.1186/s12936-017-1832-x (PMC5418711; doi:10.1186/s12936-017-1832-x)
Supplement: Supplementary file 2 — Additional file 2. Preparation of the investigational product SAR9727A (Study 1). [file 12936_2017_1832_MOESM2_ESM.docx]

**Additional file 2: Preparation of the investigational product SAR9727A**

SAR97276A was provided by Sanofi. For Study 1 SAR97276A was packed in a 1-day patient kit containing 3 vials: vial 1 contained SAR97276A 10 mg/2 mL packaged in a 36 ml vial; vial 2 contained SAR97276A 20 mg/ 2 mL packaged in a 36 ml vial; vial 3 contained phosphate solvent 3 ml packaged in a 7 ml vial. In case of the 3-day treatment, three 1-day kits were provided for each patient. For patient administration, SAR97276A concentrate was mixed with 1 mL phosphate solvent to make a neutral isotonic solution of 3.33 mg/mL or 6.67 mg/mL at pH 7.4. For IM administration this was further diluted with 0.9% NaCl in a vial. For IV administration this was further diluted in a 0.9% NaCl saline infusion bag and then given as an infusion for 30 minutes.
