# Supplementary material for: Safety and efficacy of the choline analogue SAR97276 for malaria treatment: results of two phase 2, open-label, multicenter trials in African patients
Source: Malar J. 2017 May 4;16:188. doi: 10.1186/s12936-017-1832-x (PMC5418711; doi:10.1186/s12936-017-1832-x)
Supplement: Supplementary file 3 — Additional file 3. Definition of early and late treatment failure (Study 2). [file 12936_2017_1832_MOESM3_ESM.docx]

**Additional file 3: Criteria and definition of rescue therapy (Study1 + Study 2), and definition for early and late treatment failure (Study 2)**

Study 1: In case of clinical aggravation or recrudescent parasite in an individual patient, the current available recommended rescue therapy (as per local medical guidelines) was administered.The rescue therapy is defined as any antimalarial treatment administered between first study drug intake and T72h included. Any antimalarial treatment administered after T72h is called concomitant medication.

Study 2: The patients were monitored up to day 28. In case of treatment failure (as defined below according to FDA guideline) in an individual patient, the investigator took all measures, to initiate and carry out appropriate anti-malarial treatment based on best medical practice and guidances in force. The current available recommended rescue therapy as recommended by local guidelines was given. Treatment failures were described as early and late treatment failures.

Early treatment failure was defined as development of severe malaria within the first 72 hours after first administration of study drug in the presence of parasitemia, and/or parasitemia on day 2 higher than at baseline, and/or parasitemia on day 3 with axillary temperature ≥ 37.5°C, and/or parasitemia on day 3 ≥ 25% of baseline parasitemia.

Late treatment failure was defined as development of severe malaria after day 3 after first study drug administration in presence of parasitemia, and /or parasitemia on day 4 - 14 with axillary temperature ≥ 37.5°C, and /or any additional antimalarial therapy not specified in the study protocol.
