# Supplementary material for: Safety and efficacy of the choline analogue SAR97276 for malaria treatment: results of two phase 2, open-label, multicenter trials in African patients
Source: Malar J. 2017 May 4;16:188. doi: 10.1186/s12936-017-1832-x (PMC5418711; doi:10.1186/s12936-017-1832-x)
Supplement: Supplementary file 4 — Additional file 4. Details on sample size calculation (Study 2). [file 12936_2017_1832_MOESM4_ESM.docx]

**Additional file 4: Details on sample size calculation (Study 2**)

Sample size was calculated based on the parasite reduction ratio (PRR). Standard deviations (SD) used for the calculations were estimated from Study 1. Assuming with 25 participants a SD of 0.275, the PRR was estimated with a maximum imprecision of 17.2% with 90% assurance. Therefore, if the observed ratio was 5, the 95% CI will not be wider than 4.1-6.0 with 90% assurance. To ensure 25 evaluable participants, 30 participants had to be included in each cohort.
