# Supplementary material for: Safety and efficacy of the choline analogue SAR97276 for malaria treatment: results of two phase 2, open-label, multicenter trials in African patients
Source: Malar J. 2017 May 4;16:188. doi: 10.1186/s12936-017-1832-x (PMC5418711; doi:10.1186/s12936-017-1832-x)
Supplement: Supplementary file 5 — Additional file 5. Results of hematology, electrocardiography and vital signs (Study 1). Table S1. Electrocardiogram abnormalities during TEAE period (Study1). [file 12936_2017_1832_MOESM5_ESM.docx]

**Additional file 5: Results of hematology, electrocardiography and vital signs (Study 1)**

Low hemoglobin and hematocrit values were frequently observed, especially in the children`s cohort who had hematocrit below 0.37 v/v (male), 0.32 v/v (female). This was likely caused by malaria. Alkaline phosphatase was elevated (> 1.5 x ULN) in 19 participants. Apart from the patient mentioned with an SAE due to increased transaminases, only one more patient in the adult (single IM dose) Group 1A was found with an elevated AST (> 3 x ULN). No clinical significant abnormalities were seen in the ECG [see Additional Table 1].

**Additional Table 1:** Electrocardiogram abnormalities during TEAE period (Study1)

| ECG parameter |  | 1A | 1B | 1C | 1D |
| --- | --- | --- | --- | --- | --- |
| N |  | 34 | 30 | 30 | 19 |
| QTc interval |  |  |  |  |  |
| Borderline: 431-450 ms (m); 451-470 ms (f) | | 1/33 (3%) | 2/30 (7%) | 2/30 (7%) | 3/18 (17%) |
| Prolonged: > 450 ms (m); > 470 ms (f) | | 0/33 | 0/30 | 0/30 | 0/18 |
| ≥ 500 ms |  | 0/33 | 0/30 | 0/30 | 0/18 |
| QTc interval - change from baseline |  |  |  |  |  |
| Borderline: Incr. from B 30-60 ms |  | 0/33 | 0/30 | 1/30 (3%) | 0/18 |
| Prolonged: Incr. from B > 60 ms |  | 0/33 | 0/30 | 0/30 | 0/18 |

One case of bradycardia and one of tachycardia occurred in the adult Group 1A. Two patients from the children Group 1D (3 days IM) developed thrombocytopenia.
