# Supplementary material for: Safety and efficacy of the choline analogue SAR97276 for malaria treatment: results of two phase 2, open-label, multicenter trials in African patients
Source: Malar J. 2017 May 4;16:188. doi: 10.1186/s12936-017-1832-x (PMC5418711; doi:10.1186/s12936-017-1832-x)
Supplement: Supplementary file 6 — Additional file 6. ECG parameters (Study 2). Table S2. Electrocardiogram abnormalities during TEAE period (Study2). Table S3. Respiratory parameters (Study 2). [file 12936_2017_1832_MOESM6_ESM.docx]

**Additional file 6: ECG parameters (Study 2)**

Changes in the ECG parameters were more often seen in the two SAR97276A groups (Group 2A and Group 2B) than in the control Group 2C (Additional Table 2).

**Additional Table 2:** Electrocardiogram abnormalities during TEAE period (Study2)

| ECG parameter |  |  | 2A | 2B | 2C |
| --- | --- | --- | --- | --- | --- |
| N |  |  | 8 | 8 | 4 |
| QTc interval |  |  |  |  |  |
| Borderline: 431-450 ms (Boys); 451-470 ms (Girls) | | | 3/8 | 2/8 | 1/4 |
| Prolonged: > 450 ms (Boys); > 470 ms (Girls) | | | 0/8 | 2/8 | 0/4 |
| ≥ 500 ms |  |  | 0/8 | 0/8 | 0/4 |
| QTc interval - change from baseline | |  |  |  |  |
| Borderline: Incr. from B 30-60 ms | |  | 2/8 | 0/8 | 0/4 |
| Prolonged: Incr. from B > 60 ms | |  | 0/8 | 0/8 | 0/4 |

A prolonged QTc > 450 ms was only observed in Group 2B. None of the changes was regarded as clinical significant. Respiratory parameters showed variations (Additional Table 3), however, there was no indication for an untoward effect of SAR97276A.

**Additional Table 3:** Respiratory parameters (Study 2)

| Peakflow measures | | 2A | 2B | 2C |
| --- | --- | --- | --- | --- |
| N |  | 8 | 8 | 4 |
| baseline (median L/min) | | 205 | 185 | 222.5 |
| change from baseline on: | |  |  |  |
| D1 0.30 h |  | -15.0 | 2.5 | 37.5 |
| D2 24.30 h |  | 4.0 | 5.0 | 17.5 |
| D23 48.30 h |  | 45.0 | -10.0 | 15.0 |
